# Based on network pharmacology and molecular docking to predict the mechanism of Huangqi in the treatment of castration-resistant prostate cancer

**DOI:** 10.1371/journal.pone.0263291

**Published:** 2022-05-20

**Authors:** Zesen Lin, Zechao Zhang, Xuejin Ye, Min Zhu, Zhihong Li, Yu Chen, Shuping Huang

**Affiliations:** 1 The Second People’s hospital of Zhaoqing, Zhaoqing, China; 2 Ruikang Hospital Affiliated to Guangxi University of Chinese Medicine, Nanning, China; University of Minnesota Medical School Twin Cities, UNITED STATES

## Abstract

**Background:**

As a kind of traditional Chinese medicine, HQ is widely mentioned in the treatment of cancerous diseases in China, which has been proven to have a therapeutic effect on cancerous diseases, such as prostate cancer. To predict the specific mechanism of HQ in the treatment of CRPC, we will conduct preliminary verification and discussion based on a comprehensive consideration of network pharmacology and molecular docking.

**Methods:**

TCMSP was used to obtain the compounds and reach the effective targets of HQ. The targets of CRPC were reached based on GeneCards database and CTD database. GO and KEGG were utilized for the analysis of overlapping targets. The software of Openbabel was used to convert the formats of ligands and reporters. In addition, molecular docking studies were performed by using the software of Autodock Vina.

**Result:**

It can be seen from the database results that there were 87 active compounds (20 key active compounds) in HQ, and 33 targets were screened out for CRPC treatment. GO and KEGG pathway enrichment analyses identified 81 significant GO terms and 24 significant KEGG pathways. There is a difference in terms of the expression of core protein between cancer patients and healthy people. The expression of core protein in patients also has an impact on the life cycle. The results of molecular docking showed that the docking activity of drug molecules and core proteins was better.

**Conclusions:**

It is concluded from the results of this network pharmacology and molecular docking that HQ makes a multi-target and multi-biological process, and results in the multi-channel synergistic effect on the treatment of CRPC by regulating cell apoptosis, proliferation and metastasis, which still needs further verification by experimental research.

## Introduction

Prostate cancer (PCa) ranks the second among the most common malignancies diagnosed in men. It was the third largest source of cancer-related deaths across the world in 2018, with 1,276,106 new cases annually and 358,989 deaths [1]. According to the data of 2016, the incidence of prostate cancer was 6.25%, which was the fifth most prevalent cancer across the world. The number of PCa cases increased from 1.0 million in 2006 to 1.4 million in 2016. The expected population growth rate is the function of rain-on-snow (ROS), density, and age structure, with the cases correlated with population growth rate and age structure [2]. This number is likely to continue to increase with the growth and aging of the population. The incidence of prostate cancer showed a remarkable increase among Chinese between 2010 and 2014, and prostate cancer was one of the fastest growing malignancies in China, and the sixth most prevalent cancer in Chinese [3]. A majority of Chinese PCa patients develop regionally advanced disease or widespread metastases. The patients with extensive metastases who are unable to be treated with radical surgery can only receive endocrine treatment and chemotherapy [4]. The treatment methods, such as endocrine treatment and antiandrogen treatment blocking androgens produced by the adrenal glands are able to control and improve the condition of most patients. However, after the remission period (the median period of remission period is between 14 and 30 months), most patients will enter the castrate-resistant stage, and develop into castration-resistant prostate cancer (CRPC) [5]. CRPC is divided into two types, i.e., the metastatic castration-resistant prostate cancer (mCRPC) and non-metastatic castration-resistant prostate cancer (nmCRPC). The progress of metastases of nmCRPC can be delayed by using apalutamide and enzalutamide approved by the US Food and Drug Administration, but the disease will eventually develop to mCRPC [6] which is mostly treated with docetaxel, abiraterone, prednisolone, enzalutamide, cabazitaxel and radium 223. After radical prostatectomy, adverse reactions may occur, such as decreased sexual satisfaction and voiding dysfunction. Radiotherapy patients are under the risk of second cancers. Totally 93% of patients after androgen deprivation treatment (ADT) experienced a decrease in sexual desire, as well as a decrease in the quality of life and local dysfunction [7]. Long-term ADT and chemotherapy are likely to cause adverse reactions, such as fatigue, hot flashes, muscle weakness, decreased libido, neutropenia, and vomiting [8]. Chinese herbal medicine is widely utilized in adjuvant endocrine therapy. A meta-analysis shows that this method is available for the improvement of the efficacy of adjuvant endocrine therapy without adverse reactions. However, due to the lack of uniform assessment criterion and poor methodologies, the clinical application value of Chinese herbal medicine has to be explored [9]. Chinese herbal medicine HQ has been proved by many studies to have various effects, such as anti-proliferation, pro-apoptosis, improvement of immune function, prevention of tumor metastasis, etc. [10] Astragalus (HQ) injection has an effect on breast cancer cell proliferation and Akt phosphorylation [11]. In addition, Astragalus (HQ) extract inhibits the destruction of gastric cancer cells into mesothelial cells through anti-apoptosis [12]. In our domestic research, the active ingredients of HQ were found to have an effect on the proliferation and apoptosis of prostate cancer cell line PC3 cells, and be able to inhibit the proliferation of prostate cancer cell line PC3 cells and induce their apoptosis [13]. This indicates that HQ and its active ingredients have the effects on the regulation of proliferation and apoptosis in the treatment of cancer diseases, including prostate cancer. The Chinese medicine compound containing Huangqi shows a good effect on the treatment of CRPC, prolonging the survival period of patients, and improving symptoms and the quality of life. On the other hand, the Chinese medicine compound mentioned above can also increase the anticancer activity of docetaxel [14], therefore, we suspected that HQ has the similar effects on CRPC disease. In that case, we plan to predict the mechanism of action and target by using network pharmacology and molecular docking, so as to provide a basis for the subsequent experimental research.

## Materials and methods

### 1.1 Bioactive ingredient and target identification for Huangqi (HQ)

The Chinese Medicine Systems Pharmacology Database and Analysis Platform (TCMSP) [15] is a platform for the integration of pharmacokinetics, medicinal chemistry, and drug-target-disease networks. We followed the methods of Jing Zhang et al. 2020 [16]. Based on to the TCMSP platform (http://lsp.nwu.edu.cn/tcmsp.php), the bioactive ingredients (OB) and targets of HQ were obtained. The former refers to the rate and extent of the absorption of the drug into the body’s circulation. Drug-like properties (DL) reflect the nature of a drug with a specific functional group or the same or similar physical characteristics. Bioactive ingredients were collected under the condition of OB≥30% and DL≥0.18. After that, the corresponding molecular targets of these collected active compounds were obtained by using the same database.

### 1.2 Target prediction of HQ in the treatment of CRPC

Search for CRPC-related targets with “castration-resistant prostate cancer” as a search term by using GeneCards database (https://www.genecards.org/) and the Comparative Toxicogenomics Database (CTD, ctd.mdibl.org). Venny2.1.0 (http://bioinfogp.cnb.csic.es/tools/venny/index.html) was employed to construct over-lapping targets for CRPC treatment and bioactive ingredients of HQ, allowing the identification of targets of HQ in the treatment of CRPC.

### 1.3 Construction and topological properties of compound-target networks

Compound–target networks were constructed by using the software of Cytoscape 3.7.2. The nodes degree centrality and corresponding closeness centrality obtained from compound-target networks were topologically analyzed to identify the key compounds and targets.

### 1.4 PPI networks of overlapping targets construction

The STRING database [17] (https://string-db.org/) can be used for the analysis of the interaction between proteins. In our study, the species was limited to “Homo sapiens”, and the lowest interaction score was set to medium confidence (0.400). After obtaining the PPI networks from the STRING database, Cytoscape software was utilized for further topology analysis. Finally, the node size and colour were adjusted with the software of Cytoscape to construct the complete PPI networks of overlapping targets, so as to clarify the key regulatory proteins functioned in the networks.

### 1.5 GO terms and KEGG pathway enrichment analysis

The Database for Annotation, Visualization, and Integrated Discovery (DAVID, https://david.ncifcrf.gov/) database [18] was utilized to perform Gene ontology (GO) and Kyoto encyclopedia of genes and genome (KEGG) pathway enrichment analysis. The GO terms were classified into three categories, i.e., biological process (BP), cellular component (CC) and molecular function (MF). The condition of *P*<0.01 was considered to indicate a statistically significant difference.

### 1.6 Immunohistochemical comparison and survival analysis of core targets

The immunohistochemical images of core targets were screened from The Human Protein Atlas (https://www.proteinatlas.org/) database, after that, the expressions of these targets in prostate tissues of cancer patients and normal people were compared. The cBioportal For Cancer Genomic (https://www.cbioportal.org/) database is used for the survival analysis to analyze the impact of changes in core targets on the prognosis of cancer patients.

### 1.7 Molecular docking simulation

Select corresponding ligands obtained from TCMSP database and receptors performed in the Protein Data Bank database (PDB, https://www.rcsb.org/) for molecular docking based on the compound-target network relationship. Respectively, the software of Openbabel and AutoDock Vina were used for chemical format conversion and molecular docking. The active sites of the cocrystal ligands were used as the pockets of receptors for molecular docking. The interaction of the compounds with the lowest binding free energy was analyzed on the Biotechnology Center of the TU Dresden (BIOTEC, https://projects.biotec.tu-dresden.de) platform [19–21].

### 1.8 Ethics statement

This study does not involve humans and animals. The ethical approval is not applicable to this study and this study does not need the informed consent.

## Results

### 2.1 Collection and screening of candidate active compounds in HQ

The molecular structure of each active compound was confirmed based on the TCMSP database, and then 87 compounds of HQ were retrieved. According to the criteria of OB≥30% and DL≥0.18, a total of 20 chemical ingredients were selected (as shown in Table 1).

**Table 1:** Active compounds in HQ.

Mol ID	Molecule Name	OB (%)	DL
MOL000398	isoflavanone	109.99	0.3
MOL000378	7-O-methylisomucronulatol	74.69	0.3
MOL000392	formononetin	69.67	0.21
MOL000433	FA	68.96	0.71
MOL000438	(3R)-3-(2-hydroxy-3,4-dimethoxyphenyl)chroman-7-ol	67.67	0.26
MOL000380	(6aR,11aR)-9,10-dimethoxy-6a,11a-dihydro-6H-benzofurano[3,2-c]chromen-3-ol	64.26	0.42
MOL000211	Mairin	55.38	0.78
MOL000371	3,9-di-O-methylnissolin	53.74	0.48
MOL000239	Jaranol	50.83	0.29
MOL000354	isorhamnetin	49.6	0.31
MOL000439	isomucronulatol-7,2’-di-O-glucosiole	49.28	0.62
MOL000417	Calycosin	47.75	0.24
MOL000098	quercetin	46.43	0.28
MOL000422	kaempferol	41.88	0.24
MOL000374	5’-hydroxyiso-muronulatol-2’,5’-di-O-glucoside	41.72	0.69
MOL000442	1,7-Dihydroxy-3,9-dimethoxy pterocarpene	39.05	0.48
MOL000296	hederagenin	36.91	0.75
MOL000379	9,10-dimethoxypterocarpan-3-O-β-D-glucoside	36.74	0.92
MOL000033	(3S,8S,9S,10R,13R,14S,17R)-10,13-dimethyl-17-[(2R,5S)-5-propan-2-yloctan-2-yl]-2,3,4,7,8,9,11,12,14,15,16,17-dodecahydro-1H-cyclopenta[a]phenanthren-3-ol	36.23	0.78
MOL000387	Bifendate	31.1	0.67

### 2.2 Screening of overlapping targets

**Fig 1:**
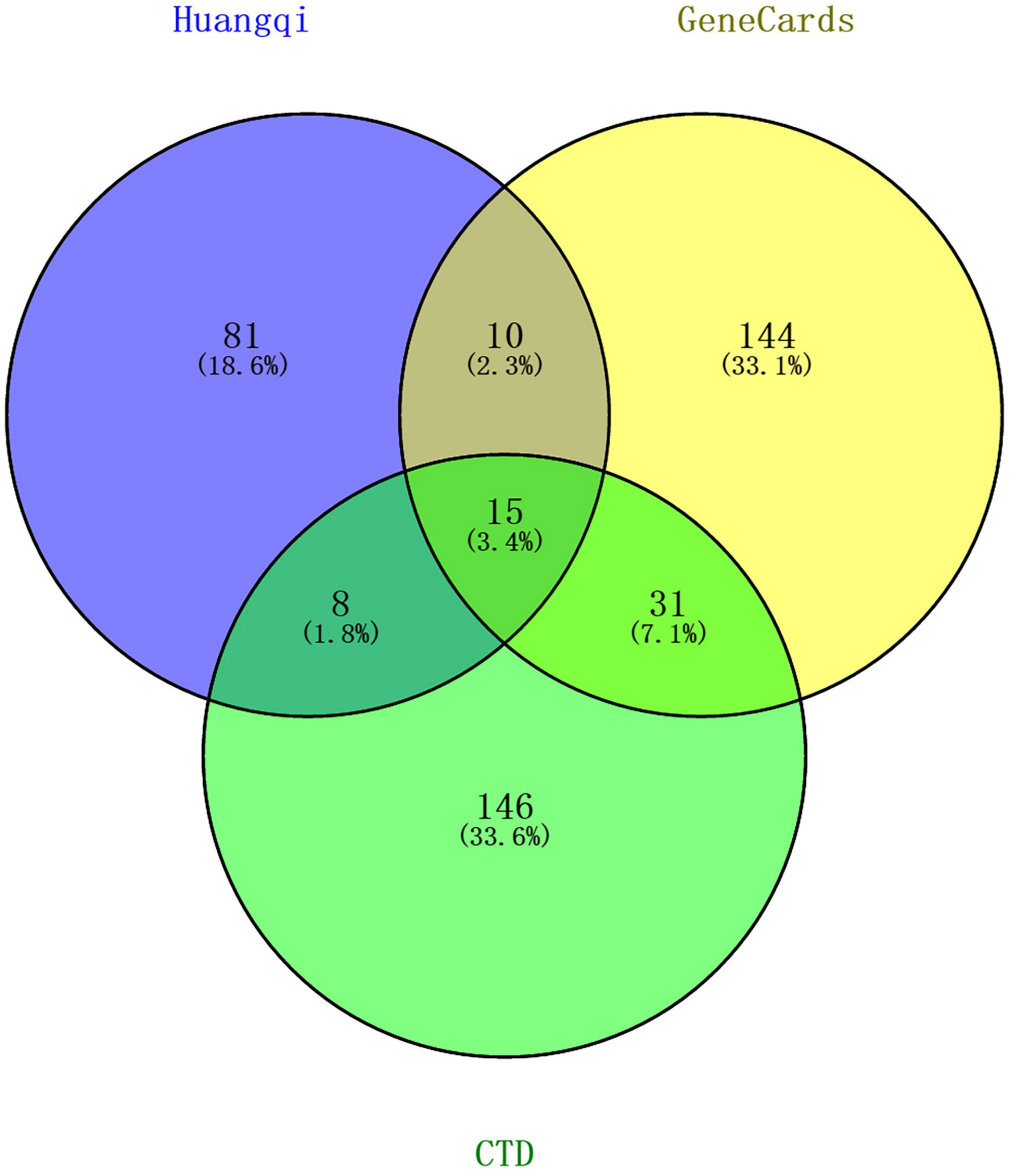
Venny diagram. 33 overlapped target genes were from three databases (TCMSP, CTD and GeneCards).

CTD and GeneCards databases were employed for the prediction of the potential targets for CRPC. Totally 2294 target genes from the CTD database and 1213 target genes from the GeneCards database were verified to be involved in CRPC. According to “Inference Score” and “Relevance score”, the top 200 results with the highest correlation in the CTD database and the top 200 results in Genecards database were obtained, respectively. 114 corresponding targets of active compounds in HQ were screened by using TCMSP database. The targets obtained above were uploaded to the Venny2.1.0 website, then the 33 overlapping targets were confirmed (as shown in Fig 1).

### 2.3 Construction of the compound–target network of HQ and CRPC

**Fig 2:**
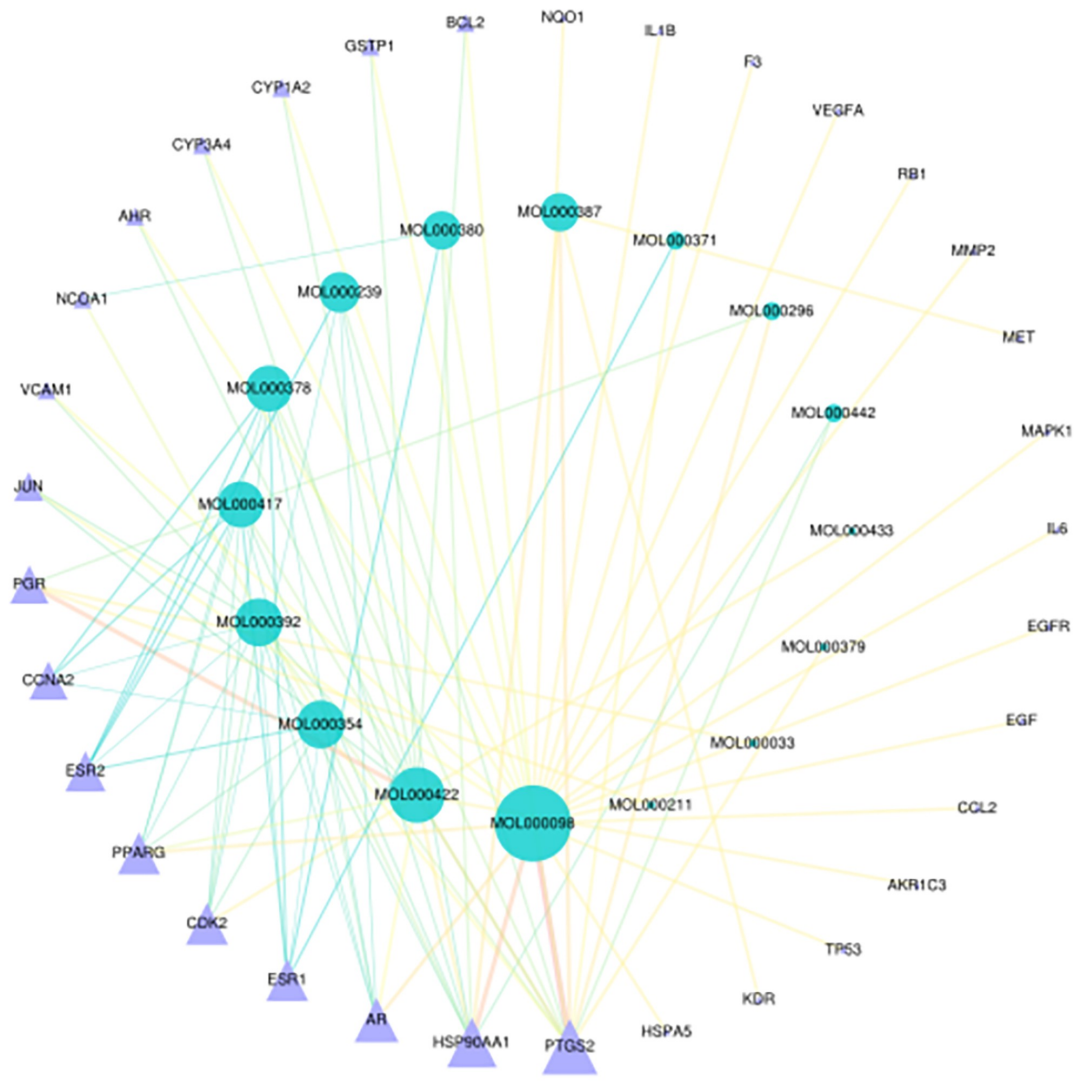
Compound–target network. The Compound-target network of HQ. The blue circle is the compound and the purple triangle is the target protein.

By analyzing the overlapping targets, only 16 compounds were found to be related to CRPC. After importing data into Cytoscape, a compound–target network was constructed (as shown in Fig 2). The parameters of the core compounds and targets involved were shown in Table 2.

**Table 2:** Active compounds in HQ.

Mol ID	Molecule Name	OB (%)	DL
MOL000398	isoflavanone	109.99	0.3
MOL000378	7-O-methylisomucronulatol	74.69	0.3
MOL000392	formononetin	69.67	0.21
MOL000433	FA	68.96	0.71
MOL000438	(3R)-3-(2-hydroxy-3,4-dimethoxyphenyl)chroman-7-ol	67.67	0.26
MOL000380	(6aR,11aR)-9,10-dimethoxy-6a,11a-dihydro-6H-benzofurano[3,2-c]chromen-3-ol	64.26	0.42
MOL000211	Mairin	55.38	0.78
MOL000371	3,9-di-O-methylnissolin	53.74	0.48
MOL000239	Jaranol	50.83	0.29
MOL000354	isorhamnetin	49.6	0.31
MOL000439	isomucronulatol-7,2’-di-O-glucosiole	49.28	0.62
MOL000417	Calycosin	47.75	0.24
MOL000098	quercetin	46.43	0.28
MOL000422	kaempferol	41.88	0.24
MOL000374	5’-hydroxyiso-muronulatol-2’,5’-di-O-glucoside	41.72	0.69
MOL000442	1,7-Dihydroxy-3,9-dimethoxy pterocarpene	39.05	0.48
MOL000296	hederagenin	36.91	0.75
MOL000379	9,10-dimethoxypterocarpan-3-O-β-D-glucoside	36.74	0.92
MOL000033	(3S,8S,9S,10R,13R,14S,17R)-10,13-dimethyl-17-[(2R,5S)-5-propan-2-yloctan-2-yl]-2,3,4,7,8,9,11,12,14,15,16,17-dodecahydro-1H-cyclopenta[a]phenanthren-3-ol	36.23	0.78
MOL000387	Bifendate	31.1	0.67

### 2.4 Constructing PPI network of the overlapping targets

**Fig 3:**
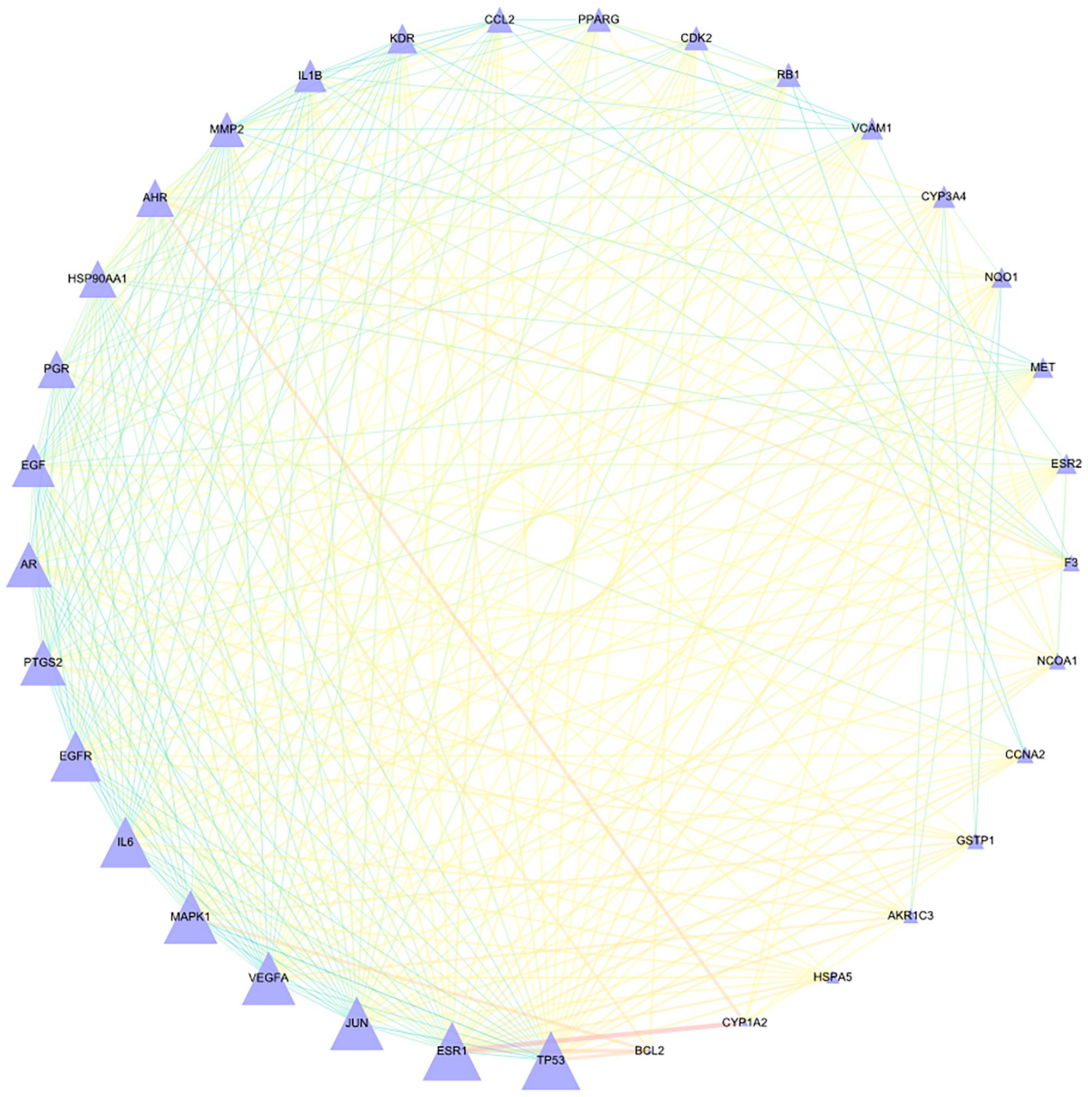
PPI network. 33 overlapped target genes were used to constructed PPI network and hub genes in PPI network. The purple triangle is the target protein. The size of the triangle represents the weight of the target protein.

The STRING database and the software of Cytoscape were used for the construction of the PPI network (as shown in Fig 3). The size and colour of the nodes represent the degree value, and the larger the size and the darker the colour, the greater the relative degree of the node. "Edge" represents the combined score, and the thicker edge represents the greater combined score. These nodes include AHR, CDK2, ESR1, ESR2, HSP90AA1, HSPA5, KDR, PGR, PPARG, TP53, AKR1C3, AR, BCL2, CCL2, EGF, EGFR, GSTP1, IL6, JUN, MAPK1, MET, MMP2, PTGS2, RB1, VEGFA, CCNA2, CYP1A2, CYP3A4, F3, IL1B, NCOA1, NQO1 and VCAM1.

### 2.5 Go and KEGG pathway enrichment analysis

**Fig 4:**
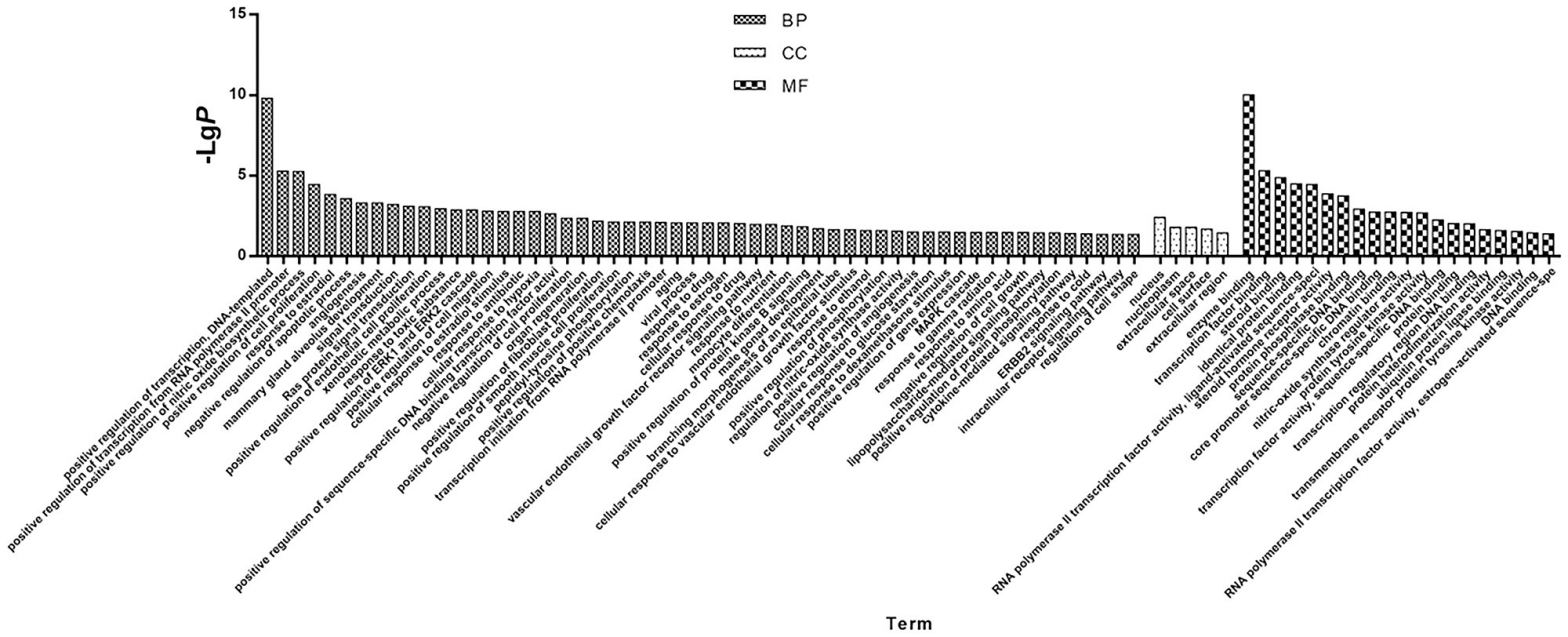
GO. 33 overlapped genes were analysis by GO annotation. BP/CC/MF are all shown in the figure, the specific meaning can be seen in the text under the bar graph.

**Fig 5:**
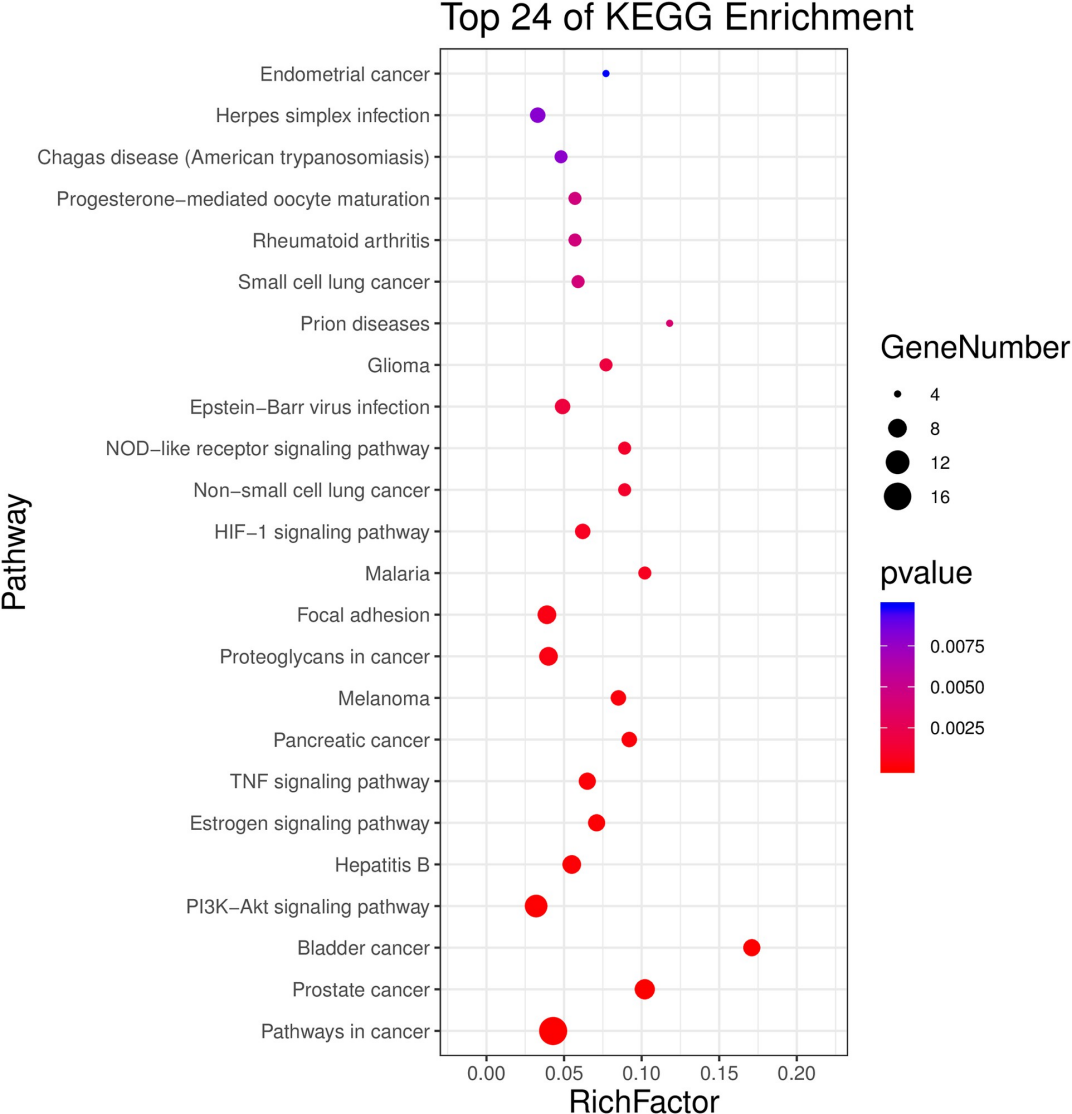
KEGG. 33 overlapped genes was analysis by KEGG, which enriched in 24 pathways. The darker the color, the greater the weight, and the larger the circle, the greater the genenumber.

**Fig 6:**
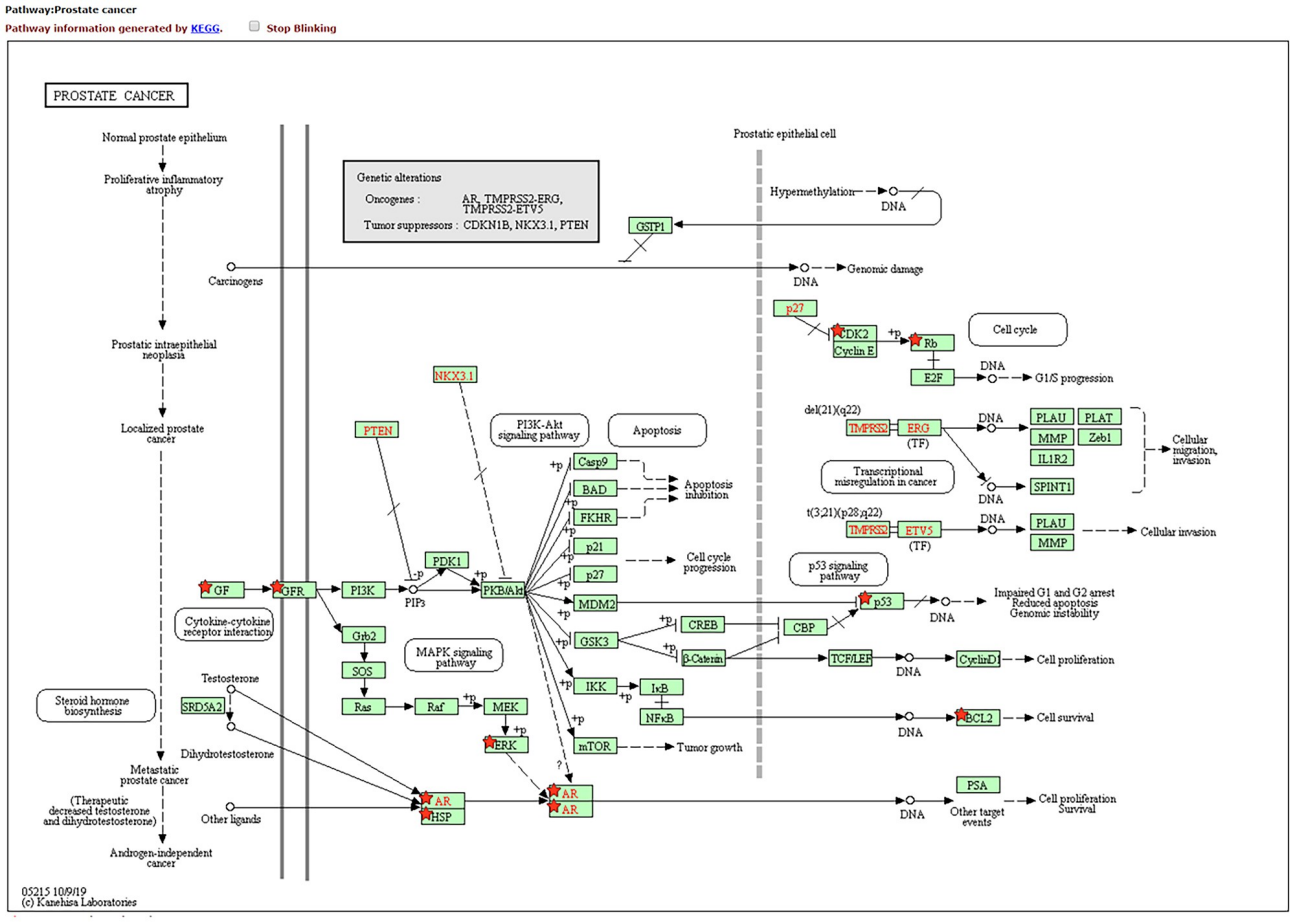
Prostate cancer pathway. Prostate cancer pathway information generated by KEGG. The highlighted part of the pathways and protein nodes related to this research.

We performed Go and KEGG enrichment analysis by using the DAVID database. Totally 20 items related with Biological process (BP), 5 items related with Molecular function (MF) and 56 items related with Cellular component (CC) were obtained, after Go enrichment analysis (p-value<0.05) (as shown in Fig 4 and Table 3). After that, 24 pathways were obtained after KEGG enrichment analysis (p-value<0.01). We uploaded the result of KEGG enrichment analysis to Omicshare (https://www.omicshare.com/) website, and then we got the Advanced Bubble Chart (Fig 5). The path of prostate cancer model in KEGG was shown in the figure (Fig 6).

**Table 3:** GO.

Type	Name
MF	enzyme binding
MF	transcription factor binding
MF	steroid binding
MF	identical protein binding
MF	RNA polymerase II transcription factor activity, ligand-activated sequence-specific DNA binding
MF	steroid hormone receptor activity
MF	protein phosphatase binding
MF	sequence-specific DNA binding
MF	core promoter sequence-specific DNA binding
MF	chromatin binding
MF	nitric-oxide synthase regulator activity
MF	protein tyrosine kinase activity
MF	transcription factor activity, sequence-specific DNA binding
MF	protein binding
MF	transcription regulatory region DNA binding
MF	protein heterodimerization activity
MF	ubiquitin protein ligase binding
MF	transmembrane receptor protein tyrosine kinase activity
MF	DNA binding
MF	RNA polymerase II transcription factor activity, estrogen-activated sequence-specific DNA binding
CC	nucleus
CC	nucleoplasm
CC	extracellular space
CC	cell surface
CC	extracellular region
BP	positive regulation of transcription, DNA-templated
BP	positive regulation of transcription from RNA polymerase II promoter
BP	positive regulation of nitric oxide biosynthetic process
BP	positive regulation of cell proliferation
BP	response to estradiol
BP	negative regulation of apoptotic process
BP	angiogenesis
BP	mammary gland alveolus development
BP	signal transduction
BP	Ras protein signal transduction
BP	positive regulation of endothelial cell proliferation
BP	xenobiotic metabolic process
BP	response to toxic substance
BP	positive regulation of ERK1 and ERK2 cascade
BP	positive regulation of cell migration
BP	cellular response to estradiol stimulus
BP	response to antibiotic
BP	cellular response to hypoxia
BP	positive regulation of sequence-specific DNA binding transcription factor activity
BP	negative regulation of cell proliferation
BP	organ regeneration
BP	positive regulation of fibroblast proliferation
BP	positive regulation of smooth muscle cell proliferation
BP	peptidyl-tyrosine phosphorylation
BP	positive regulation of positive chemotaxis
BP	transcription initiation from RNA polymerase II promoter
BP	aging
BP	viral process
BP	response to drug
BP	response to estrogen
BP	cellular response to drug
BP	vascular endothelial growth factor receptor signaling pathway
BP	response to nutrient
BP	monocyte differentiation
BP	positive regulation of protein kinase B signaling
BP	male gonad development
BP	branching morphogenesis of an epithelial tube
BP	cellular response to vascular endothelial growth factor stimulus
BP	response to ethanol
BP	positive regulation of phosphorylation
BP	regulation of nitric-oxide synthase activity
BP	positive regulation of angiogenesis
BP	cellular response to glucose starvation
BP	cellular response to dexamethasone stimulus
BP	positive regulation of gene expression
BP	MAPK cascade
BP	response to gamma radiation
BP	response to amino acid
BP	negative regulation of cell growth
BP	lipopolysaccharide-mediated signaling pathway
BP	positive regulation of protein phosphorylation
BP	cytokine-mediated signaling pathway
BP	response to cold
BP	ERBB2 signaling pathway
BP	intracellular receptor signaling pathway
BP	regulation of cell shape

### 2.6 Immunohistochemistry and survival analysis

**Fig 7:**
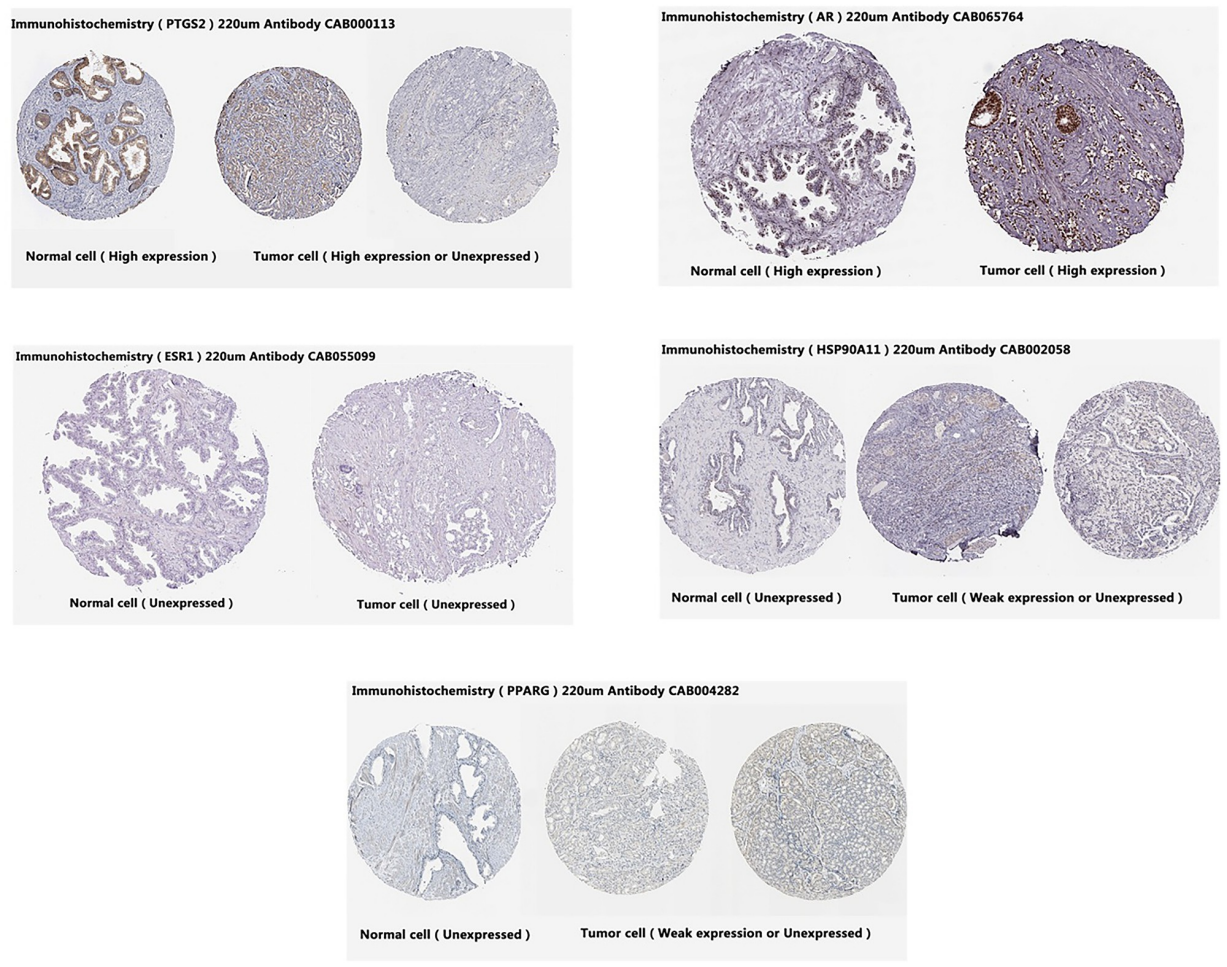
Immunohistochemistry. The expressions of 5 targets in prostate tissues of cancer patients and normal people.

**Fig 8:**
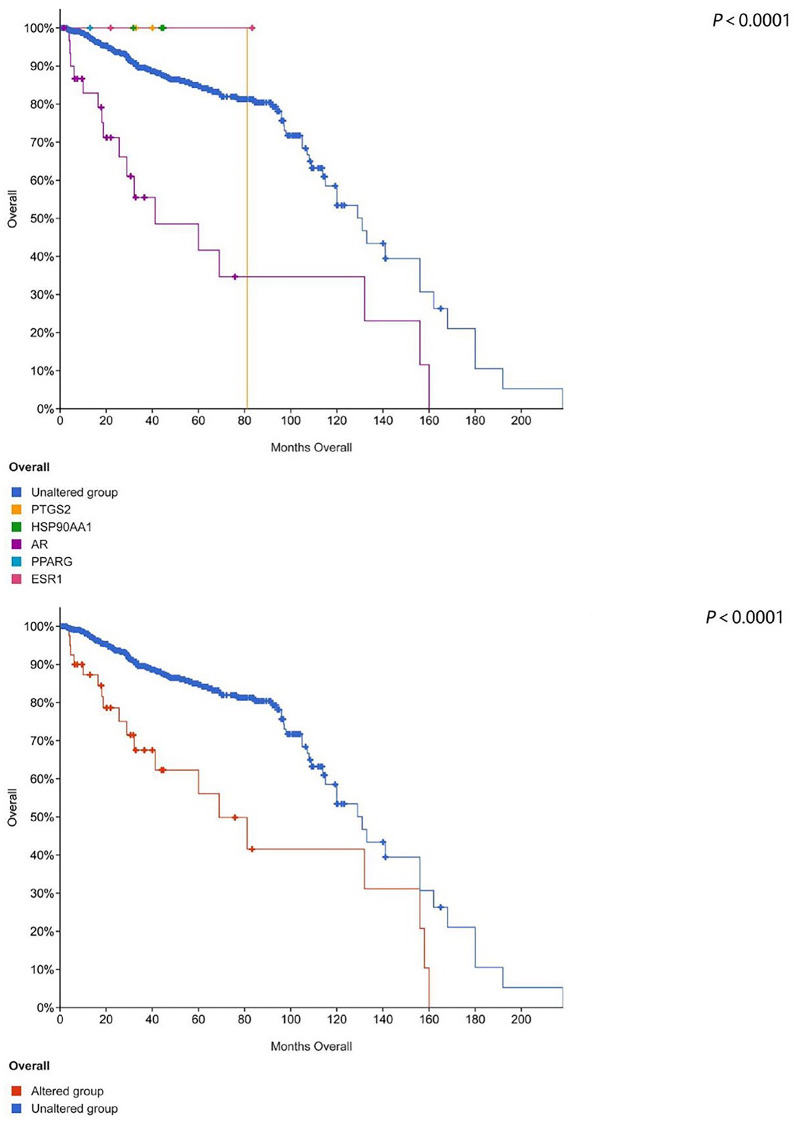
Survival analysis of five core proteins. Survival analysis involves five core proteins. The blue line in the figure indicates the survival of prostate cancer patients whose targets had not changed. Non-blue lines show changes in the survival of prostate cancer patients with changed targets.

**Fig 9:**
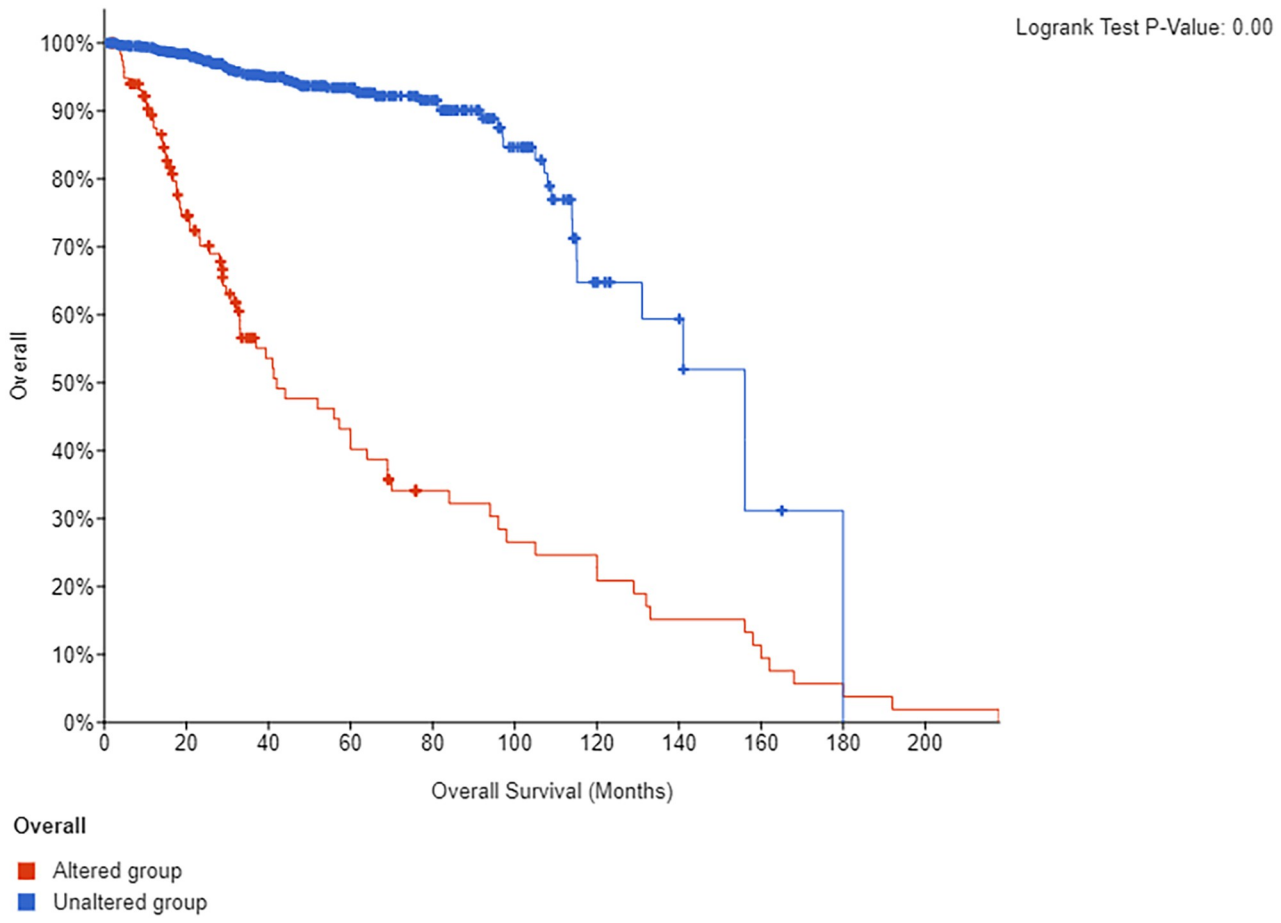
Survival analysis of AR. Survival analysis involves AR gene. The blue line in the figure indicates the survival of prostate cancer patients whose targets had not changed. Non-blue lines show changes in the survival of prostate cancer patients with changed targets.

The Human Protein Atlas database showing the expressions of these targets in prostate tissues of cancer patients and normal people is different. Prostate tumor and normal prostate tissues show elevated AR expression instead of ESR1 expression. Though normal prostate tissues show no HSP90AA1 expression, in prostate tumor tissues, the gene is weakly expressed or not expressed. PPARG gene is not expressed in normal prostate tissues but weakly expressed or not expressed in prostate tumor tissues. Normal prostate tissues show elevated PTGS2 expression, but prostate tumor tissues show elevated expression or no expression (as shown in Fig 7). It can be seen from the results that most of these core proteins change in prostate tissue after having cancer. We further used the existing cancer database (https://www.cbioportal.org/) for survival analysis for five core proteins (as shown in Fig 8). The blue line in the figure indicates the survival of prostate cancer patients whose targets have not changed. These prostate cancer patients have survived for more than 200 months. Non-blue lines show the changes in the survival of prostate cancer patients with changed targets. The results show that regardless of the effect of a single target or five core targets, the average survival of patients is less than 160 months (*P*<0.0001). We performed a survival analysis of the AR gene using cancer database (https://www.cbioportal.org/) (Fig 9) (Querying **6875** patients / **7161** samples in 22 studies). It can be seen from the results that whether the AR gene is changed or not has a significant impact on the life cycle of Pa patients (*P*<0.05).

### 2.7 Molecular docking simulation

The top five targets in Degree of PPI network were utilized for molecular docking. The molecular docking scores of PTGS2, HSP90AA1, AR, PPARG and ESR1 are as follows (as shown in Table 4). According to the results, the active compounds could produce active binding with the core targets.

**Table 4:** Molecular docking.

Target name	PDB ID	MOL	Vina score
AR	1E3G	MOL000098	-8
		MOL000239	-6
		MOL000354	-6.9
		MOL000378	-4.9
		MOL000392	-5.4
		MOL000417	-5.7
		MOL000422	-8.8
ESR1	4ZNH	MOL000354	-8
		MOL000371	-8.4
		MOL000378	-7.7
		MOL000380	-9.2
		MOL000392	-9.3
		MOL000417	-8.9
HSP90AA1	5FWK	MOL000098	-9.3
	5FWL	MOL000239	-8.5
	5FWL	MOL000354	-9.3
	5FWL	MOL000378	-9
	5FWL	MOL000380	-9
	5FWK	MOL000387	-8.2
	5FWK	MOL000392	-8.5
	5FWL/5FWK	MOL000417	-9
	5FWL	MOL000422	-8.9
	5FWL	MOL000442	-9.2
PPARG	1FM6	MOL000098	-8.5
		MOL000354	-8
		MOL000378	-7.9
		MOL000392	-8.4
		MOL000417	-8.3
		MOL000422	-8.7
PTGS2	5F19	MOL000098	-9
		MOL000239	-8.1
		MOL000296	-8.6
		MOL000354	-9.2
		MOL000371	-8.7
		MOL000378	-8.1
		MOL000379	-9.6
		MOL000380	-8.5
		MOL000387	-6
		MOL000392	-8.2
		MOL000417	-9.1
		MOL000422	-9.4
		MOL000442	-8.9

**Fig 10:**
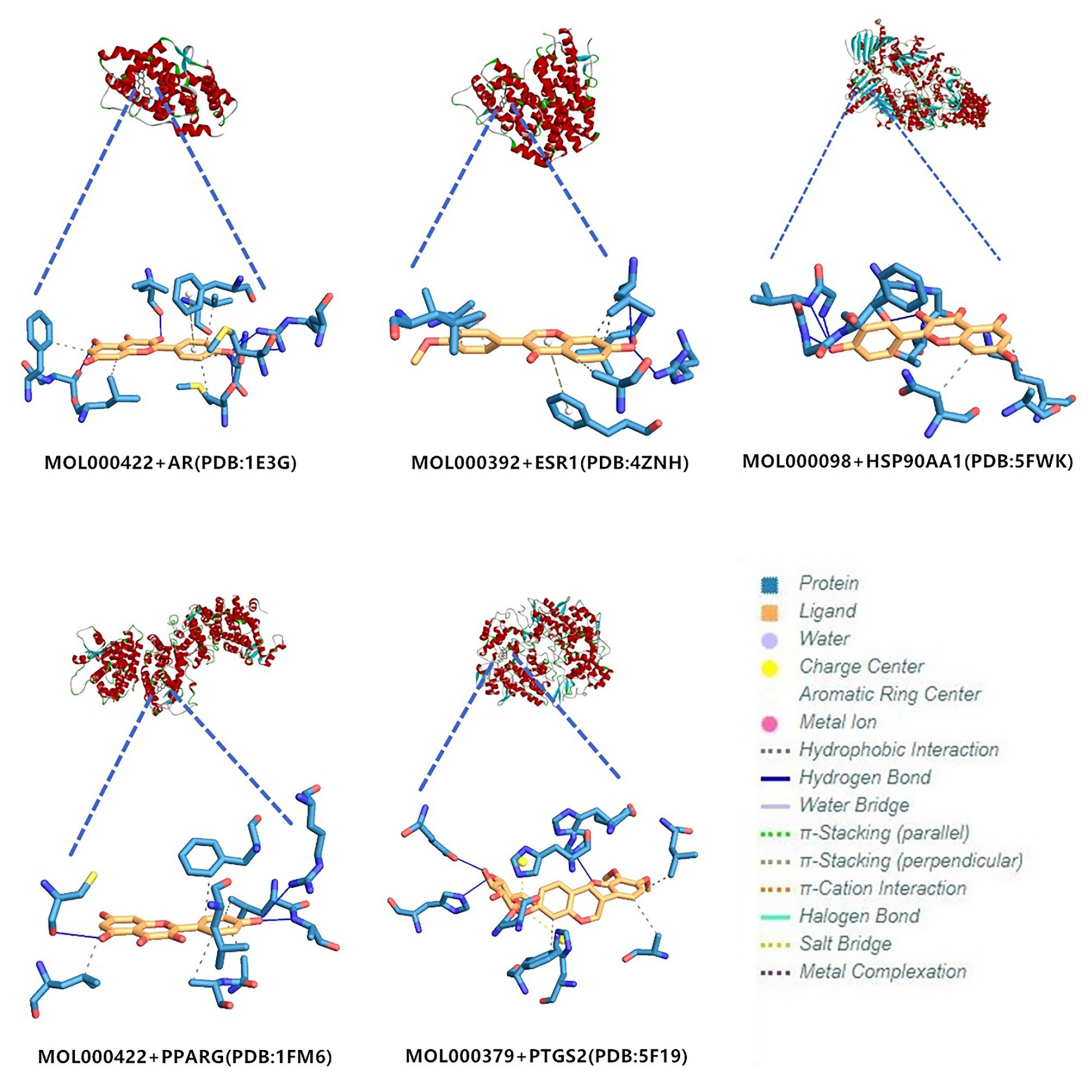
Core targets molecular docking. Core targets molecular docking. The meaning of colors and graphics can be known from the legend.

We selected the compounds with the lowest score of Vina in the docking (as shown in Fig 10) for further analysis. The results show that, from the perspective of spatial structure, small drug molecules are located in the active sites where the cocrystal ligands are located. In addition, there are multiple hydrophobic interactions, atom binding site and hydrogen bonding between small drug molecules and protein residues.

## Discussion

Prostate cancer is a common malignant tumor in male genito-urinary system. There is currently no way to prevent the disease, and there are many side effects in the treatments [22]. In the United States, although most cases are diagnosed early, some cases will still manifest or proceed into metastatic diseases and eventually develop metastatic castration-resistant prostate cancer. Metastatic prostate cancer is a global disease with a high incidence, and the median survival of patients with mCRPC is less than two years with their mortality rate exceeding 50%. In contrast, few treatments are able to delay the progression of nmCRPC to mCRPC, or delay the time for patients with nmCRPC to be treated with cytotoxic chemotherapy. Although there are many drugs for the patients to choose from, the sad fact is that mCRPC is an incurable disease. The existing drugs have little effect on the survival rate of patients with CRPC [23,24], therefore, we have to find new treatments or combined treatments to improve the curative effect, and reduce the side effects of the treatment to extend the patient’s life cycle and improve the patient’s quality of life. The researches on network pharmacology and verification measures of molecular docking emerged in recent years have provided us with the possibility to find new treatments.

The objects studied in this paper include the HQ and its extracts which play an anti-tumor role through multicellular pathways in breast cancer, gastrointestinal cancer and ovarian cancer [25–27]. MOL000098 (quercetin), MOL000422 (kaempferol), MOL000442 (1,7-Dihydroxy-3,9-dimethoxy pterocarpene), MOL000417 (Calycosin), MOL000392 (formononetin), MOL000379 (9,10-dimethoxypterocarpan-3-O-β-D-glucoside), MOL000378(7-O-methylisomucronulatol), MOL000371 (3,9-di-O-methylnissolin), MOL000354 (isorhamnetin) are flavonoids which are available to inhibit the production of fatty acids in cancer cells, and have cancer cell toxicity [28,29]. The results of Liquid Chromatography Analysis suggested that flavonoids and saponins were the main active substances of HQ [30,31]. Quercetin with the effect of antitumor proliferation may cause tumor cell apoptosis by regulating mitochondrial cytochrome C, which can also inhibit the production of cancer stem cells causing cancer to recur [32]. Calycosin enhances the effect of TGF-β on apoptosis, which can inhibit the proliferation of cancer cells through WDR7-7-GPR30 signaling [33,34]. Formononetin can induce prostate cancer transformation through the ERK1/2 MAPK-Bax pathway [35], which can also inhibit the G1 cell cycle by inactivating Akt/cyclin D1/CDK4, making it exhibit inhibitory activity on human prostate cancer cells both in vivo and in vitro [36]. Isorhamnetin can selectively inhibit the PI3K–Akt–mTOR pathway, which can inhibit overexpression of matrix metalloproteinase 2 (MMP-2) and MMP-9, as well as the cell migration and invasion in a concentration-dependent manner. These findings suggest that Isorhamnetin has therapeutic potential in androgen-independent prostate cancer [37]. MOL000433 (FA) is a phenolic compound with the functions of antioxidant, antibacterial, anti-allergen, anti-inflammatory, anti-hypoglycemia, anti-pathogenicity and anti-virus. FA can reduce the expression of genes causing cell cycle arrest in the G1/S phase of prostate cancer cells by enhancing the cellular response of prostate cancer cell lines, resulting in cell cycle arrest. In experimental studies, it was found that the expression of tumor suppressor genes and apoptosis genes in prostate cancer cells after FA treatment increased significantly. On the contrary, the gene expression of anti-apoptotic protein BCL2 was significantly reduced, indicating that FA has apoptotic activity on prostate cancer cells [38]. MOL000387 (Bifendate) is also called Mairin (Betulinic acid). BA is able to prevent the growth of various human cancer cells by changing the key signaling pathways involved in apoptosis, which may induce apoptosis by stabilizing p53 in human prostate cancer cells and down-regulating the NF-κB pathway [39]. Other active compounds have not been reported to have an effect on tumor diseases. Though they may have an effect of anti-tumor, further research is still necessary. Through the analysis of prostate cancer pathways, the main pathways include P13-Aktsignal pathway, P53 and MAPK signal pathway. P13K-Aktsignal pathway is related to various cancers, such as PCa [40–43]. As one of the most important tumor suppressor genes, P53 has the potential to resist apoptosis of PCa cells, and its functional status is important in the progress of PCa. The P53 with a higher mutation rate has more mutations in advanced metastatic PCa. This mutation not only seriously destroys the function of P53 protein, but also reduces the disease-free survival of patients [44–46]. The mutation of TP53 is also active in CRPC, which is related to the poor prognosis of CRPC [47]. The RAS-MAPK signaling pathway with therapeutic potential in CRPC involves a wide range of cellular processes, including differentiation, proliferation and survival. Besides, RAS-MAPK has become a key pathway for human cancer. In many human cancers, the abnormal activation of RAS-MAPK has an important carcinogenic effect [48,49].

The core targets involved in this study include PTGS2, HSP90AA1, AR, PPARG and ESR1. PTGS2 produces inflammatory prostaglandins, and the upregulation of PTGS2 is associated with the increase of cell adhesion, phenotypic changes, resistance to apoptosis and tumor angiogenesis. PTGS2 related to the proliferation, invasion, apoptosis, host immune response and angiogenesis of malignant tumors as well as tumor radioresistance is associated with the growth and survival of PCa, which has been shown to be overexpressed in malignant tumors [50]. Increased COX-2 expression occurs in high-grade PCa [51]. HSP90AA1 is expressed highly in most cancers, but poorly in prostate cancer tissue [52]. Besides, its mechanism of action in prostate cancer has to be further studied. Almost all prostate cancer cells depend on androgen and AR signals which are closely related to prostate development. Experimental studies have shown that long-term exposure to high or low systemic androgens can increase the incidence of prostate cancer [53]. The occurrence and development of CRPC mainly depend on androgen-androgen receptor signaling pathway [54]. And 40% to 60% of mCRPC patients have AR, DNA mismatch repair, PI3K and other gene mutations [55]. By further research on the pathogenesis of CRPC, it is found that AR available to drive tumor progression is still the key factor to promote the occurrence and development of CRPC, therefore, androgen deprivation therapy remains the basic means to control the occurrence and development of CRPC [56]. In clinical investigations, it was found that the lack of PPARG (PPARγ) might be related to the development of PCa [57]. PARG and PRKAR2B genes may act as the potential biomarkers for the treatment of PCa [58]. The activity of PPARγ is related to the occurrence and development of prostate cancer. Besides, the inhibition of the expression of PPARγ may have a preventive and therapeutic effect on prostate cancer. Therefore, some scholars have identified PPARγ as an important new therapeutic target for prostate cancer [59]. ESR1 has the effects of stimulating abnormal prostate growth, controlling prostate cell growth and programming prostate cell death, and these effects are associated with prostate cancer susceptibility. Some meta-analyses suggest that ESR1 polymorphisms may increase the risk of prostate cancer in American and Indian populations [60]. These studies indicate that core targets play a role in prostate cancer and CRPC. According to the survival analysis, the survival time of prostate cancer patients with changed core targets was shortened accordingly. It can be seen that these targets play an important role in the progression of prostate cancer, and interventions to these targets may lead to a positive effect on the improvement of the prognosis of patients with prostate cancer. The results of molecular docking and interaction analysis exhibit good docking activity, therefore, the results of this molecular docking simulation are of reference value for the development of CRPC drugs.

This study reveals the pharmacological mechanism of HQ in the treatment of CRPC at the system level through network pharmacology. We speculate that the active ingredients of the drug have a curative effect on the regulation of the proliferation, apoptosis and metastasis of prostate cancer cells. Network pharmacological analysis and molecular docking verification show that HQ has a potential therapeutic effect on the treatment and control of prostate cancer, and it also has the potential to delay the late conversion of the disease into CRPC. However, the traditional Chinese medicine HQ has a multi-target and multi-level regulation effect. This research only studied the pharmacological effects on the micro level, and these results have to be further confirmed by experimental research. Due to the fact that this research is mainly carried out at the theoretical level, in the later stage, the research group will purify key compounds, explore the appropriate therapeutic concentration, and conduct animal experiments as well as clinical trials around pharmacokinetics and pharmacodynamics, thereby providing the theoretical and practical basis.

## Supporting information

S1 File Compound intersection target. (XLSX)

S2 File Dock fraction. (XLSX)

S3 File (DOCX)
