# Supplementary material for: Based on network pharmacology and molecular docking to predict the mechanism of Huangqi in the treatment of castration-resistant prostate cancer
Source: PLoS One. 2022 May 20;17(5):e0263291. doi: 10.1371/journal.pone.0263291 (PMC9122509; doi:10.1371/journal.pone.0263291)
Supplement: S3 File — (DOCX) [file pone.0263291.s003.docx]

The datasets generated and analysed during the current study are available in the “https://figshare.com/” repository, [https://doi.org/10.6084/m9.figshare.13073222.v2；https://doi.org/10.6084/m9.figshare.13073237.v1；https://doi.org/10.6084/m9.figshare.13073252.v1；](https://doi.org/10.6084/m9.figshare.13073222.v2；https:/doi.org/10.6084/m9.figshare.13073237.v1；https:/doi.org/10.6084/m9.figshare.13073252.v1；)
